# Expression of immune checkpoint receptors Indoleamine 2,3‐dioxygenase and T cell Ig and ITIM domain in metastatic versus nonmetastatic choroidal melanoma

**DOI:** 10.1002/cam4.2167

**Published:** 2019-04-16

**Authors:** Gustav Stålhammar, Stefan Seregard, Hans E. Grossniklaus

**Affiliations:** ^1^ St. Erik Eye Hospital Stockholm Sweden; ^2^ Department of Clinical Neuroscience Karolinska Institutet Stockholm Sweden; ^3^ Departments of Ophthalmology and Pathology Emory University School of Medicine Atlanta Georgia

**Keywords:** digital image analysis, IDO, immune checkpoints, immunohistochemistry, TIGIT, Uveal melanoma

## Abstract

**Background:**

Survival in metastasized cutaneous melanoma (CM) has been improved with the advent of inhibitors of immune checkpoints CTLA4 and PD‐1. In contrast, the response rate for inhibition of these checkpoints in uveal melanoma (UM) is very low. Other checkpoints including IDO and TIGIT may be targetable.

**Methods:**

Sections from 6 patients with UM, who had undergone primary enucleation 1978—1995 and 6 paired liver metastases were stained immunohistochemically (SOX10, Melan‐A, IDO, TIGIT, and CD8). Four tumors from patients who did not develop metastasis during a mean follow‐up of 19 years, and 5 samples each of normal choroidal and liver tissue were included for comparison. The number of cells/mm^2^ expressing IDO, TIGIT and CD8 was counted with manual and digital image analysis methods. Retrospective data on patient and tumor characteristics was reviewed.

**Results:**

The number of TIGIT positive cells was significantly higher in primary tumors from patients who eventually developed metastases (mean 4695 cells/mm^2^) than from patients who didn't (mean 1342 cells/mm^2^, *P* < 0.01) and paired metastases (463 cells/mm^2^, *P* < 0.01). The number of IDO positive cells was not significantly higher in metastatic tumors (*P* = 0.079), but the number of IDO and TIGIT positive cells/mm^2^ correlated in both hot spots (*R* ^2^ = 0.24, *P* < 0.01) and full tumor sections (*R* ^2^ = 0.35, *P* < 0.01).

**Conclusion:**

The expression of immune checkpoint receptor TIGIT is increased in primary uveal melanomas that seed metastases, and correlates with the expression of checkpoint receptor IDO. Both may be future targets for therapy.

## 1 INTRODUCTION

Uveal melanoma (UM) is the most common primary intraocular tumor in adults, with a propensity for liver metastasis.^1^ Although there are several well‐established options for treatment of the primary tumor, there is no effective therapy for metastatic disease.

Survival in metastasized cutaneous melanoma (CM) has been greatly improved with the advent of kinase inhibitors for BRAF‐mutant tumors and monoclonal antibodies against the cytotoxic T‐lymphocyte‐associated protein 4 (CTLA4) and Programmed cell death protein 1 (PD‐1) immune checkpoints.^2^ Used as single agents, response rates of 10%‐20% for anti CTLA4 and 40% for anti‐PD‐1 have been reported. In combination, the response rate is as high as 44%, with a progression‐free survival (PFS) of 11.5 months.^3^

For UM however, single agent response rates are approximately 5% and in combination 0%‐17%, with a progression‐free survival of less than 3 months.^4^, ^5^

The difference between metastasized UM and CM in effect of checkpoint inhibition is likely a consequence of the difference in mutational load and immunogenicity. UM lack BRAF‐mutations and has a markedly low mutation rate, as shown by the Cancer Genome Atlas (TCGA).^6^, ^7^ Expression of the PD‐1 ligand (PD‐L1) as determined by immunohistochemistry is significantly lower than in CM.^8^, ^9^ Tumor‐infiltrating lymphocytes isolated from UM are also less expansive ex vivo in response to exogenous IL‐2, reflecting a lower proliferative capacity than their CM counterpart.^9^

Patients hoping to benefit from the great potential of immunotherapy may however direct their attention to evidence that coinhibitory checkpoints are active in UM. CD8 positive T cell infiltrates have been identified in a third of primary UM with high risk for metastasis, based on monosomy 3 (M3), but not in tumors with disomy 3 (D3).^7^ In DNA methylation and RNA‐sequencing analysis, tissue from the M3 tumors were found to overexpress the immune checkpoints* Indoleamine 2,3‐dioxygena*se (*IDO1*) and *T cell Ig and ITIM domain* (*TIGIT*) genes.^7^ *IDO1* encodes the heme‐containing IDO protein, that contributes to metabolic immune regulation by catalyzing catabolism of tryptophan along the kynurenine pathway, which limits T‐cell function and engage mechanisms of immune tolerance.^10^, ^11^ *TIGIT* encodes an immune receptor on T cells, regulatory T cells, and Natural Killer cells (NK) that decreases cell proliferation, cytokine production, and degranulation.^12^, ^13^ TIGIT shares ligands with the T cell and NK receptors CD96 and CD226, and together form a pathway that closely resembles CTLA4.^14^

Blockade of IDO and TIGIT with monoclonal antibodies increases the proportion of antitumoral T cells and delays tumor growth in vitro*.* 11, 13 Several clinical studies with IDO and TIGIT‐inhibitors are planned or underway. Mostly, these recruit patients with other cancers and no results on response rates or survival for patients with UM have been presented.

The potential for targetable immune checkpoints in UM is encouraging. Here, we seek to add to the previous findings in primary tumors by investigating the immunohistochemical expression of IDO and TIGIT in primary tumors and paired metastases from patients with very long (>30 years) follow‐up. Primary tumors from patients that did not develop metastasis are included for comparison.

## 2 MATERIALS AND METHODS

### 2.1 Patients and samples

The study adhered to the tenets of the Declaration of Helsinki. The protocol for collection of specimens and data was approved by the regional ethical review board in Stockholm, Sweden. Formalin fixed paraffin embedded (FFPE) eyes with UM in the posterior choroid from 6 deceased patients that had undergone primary enucleation without previous plaque brachytherapy, external beam radiation or transpupillary thermotherapy at St. Erik Eye Hospital between the years 1971 and 1995 were collected along with FFPE incisional biopsies from their liver metastases obtained during autopsy at Karolinska University Hospital between the years 1978 and 1997. For comparison, FFPE eyes with posterior choroidal melanoma were collected from 4 deceased patients that had undergone primary enucleation at St. Erik Eye Hospital in 1984 and 1985 without developing metastasis before death, as well as 5 pieces of normal choroid and liver tissue. Plaque brachytherapy has been the treatment of choice for tumors with an apical height of <6‐7 mm since 1979, but eyes such as the ones included in this study can still be enucleated based on patient preference, tumor location and other risk factors. Clinicopathological data on age at enucleation, gender, tumor location and extension, AJCC T‐category,^15^ tumor thickness, diameter, previous treatments, time, and cause of death were collected from the records of the Oncology and Pathology service, St. Erik Eye Hospital.

### 2.2 Immunohistochemistry

The paraffin blocks were cut into 4 μm sections at the laboratory of the Oncology and Pathology service, St. Erik Eye Hospital, Stockholm, Sweden and then sent to the LF Montgomery Ophthalmic Pathology Laboratory, Emory Eye Center, Atlanta, GA, USA. Sections were then pretreated in EDTA‐buffer at pH 9.0 for 20 minutes and incubated with mouse monoclonal antibodies against IDO (catalog no. 05‐840, Sigma‐Aldrich, Saint Louis, MO, USA), CD8 (catalog no. GA62361‐2, Agilent Technologies Inc Santa Clara, CA, USA) and Melan‐A (catalog no. M719601‐2, Agilent), and with rabbit polyclonal antibodies against TIGIT (catalog no. ab233404, Abcam, Cambridge, UK) and SOX10 (catalog no. ab108408, Abcam) according to the manufacturers’ instructions. A red chromogen was used. Sections were finally counterstained with hematoxylin and rinsed with deionized water. Tonsil tissue was used as positive control in gradually titrated concentrations of IDO, TIGIT, and CD8 until optimal staining was achieved according to manual supervision by a pathologist (GS). The deparaffinization, pretreatment, primary staining, secondary staining, and counterstaining steps were run in a Bond III automated IHC/ISH stainer (Leica, Wetzlar, Germany).

### 2.3 Density of cells expressing IDO, TIGIT, and CD8

We counted the number of stained cells in tumors’ hot spots as well as across full tumor sections, expressed as the number of IDO, TIGIT, and CD8 positive cells/mm^2^. In assessments of hot spots, tissue sections were first screened at 40×, and the area with the most intense staining was selected. In this area, the number of positive cells (IDO and TIGIT cytoplasmic stain, CD8 cell membranous stain) per 3 high power fields (hpf) was counted at 400×, corresponding to a field diameter of 0.5 mm per hpf, and an aggregated area over 3 hpf of 0.6 mm^2^. To allow for consistent detection of stained cells among the up to several millions of cells across a full tumor section, all glass slides were also digitally scanned at ×400, using a Nano Zoomer 2.0 HT (Hamamatsu Photonics KK, Hamamatsu, Japan) at the Winship Core Pathology Laboratory, Winship Cancer institute of Emory University. The number of positive cells per mm^2^ of tumor tissue was defined in primary tumor and metastases with the QuPath Bioimage analysis software, v. 0.1.2, under supervision by a pathologist (GS).^16^ A positive stain vector (Red chromogen marking IDO, TIGIT, or CD8 expression) and negative stain vector (haematoxylin in tumor cell nucleus) was defined in each tissue section, and a region of interest drawn along the tumor's margins. All nontumor tissues were excluded, as well as tumor areas with intense inflammation, abundant pigmentation, fibrosis, bleeding, necrosis, tissue folds, or poor fixation. The “positive cell detection” function was then run with the settings described in Table S1. All operations were performed blinded to patient identities and outcomes. The software was run on a standard off‐the‐shelf lap top computer (Apple, Inc Cupertino, CA).

### 2.4 Statistical methods

We compared the mean number of IDO, TIGIT, and CD8 positive cells in hot spots and full tumor sections/mm^2^ in primary tumors from patients that did and did not develop metastasis before death, as well as in metastases and in normal choroid and liver tissue. No previous papers comparing the proportions of IDO and TIGIT positive cells between metastatic and nonmetastatic tumors were available for basing power calculations on. In a post‐hoc analysis with patients that did not develop metastasis before death as the reference group, assuming a 2‐sided α of 0.05, we would be able to detect differences of 46% and 34% with a power of 0.80 for IDO and TIGIT, respectively. Differences with a *P* < 0.05 were considered significant, all p‐values being 2‐sided. The deviation from normal distribution was not statistically significant for any continuous variable, when evaluated by the Shapiro–Wilk test (*P* > 0.05). All variances were equal, with the exception of follow‐up years between patients that did and did not develop metastasis before death (Levene's test for equality of variances *P* > 0.05). For tests of all other continuous variables, we therefore used Student's *t* test with equal variances assumed. Fisher's exact test was used for two‐by‐two tables. Linear regression correlations of numerical values of IDO, TIGIT and CD8 positive cells/mm^2^ in different tissues were calculated, with statistical significance tested with one‐way ANOVA. For correlation to outcome, IDO and TIGIT expression were evaluated with Cox regression for association with metastasis. All statistical analyses were performed using IBM SPSS statistics version 25 (Armonk, NY).

## 3 RESULTS

### 3.1 Descriptive

Of our 10 patients, 5 were men and 5 were women. The mean age at enucleation was 64 years (SD 14). No patient had been treated with plaque brachytherapy, external beam radiation or transpupillary thermotherapy. All primary tumors (*n* = 10) were of AJCC T‐Category 2 and located in the choroid posterior to the equator of the eye, without ciliary body involvement, extrascleral extension, optic nerve or vortex vein invasion.

**Figure 1:**
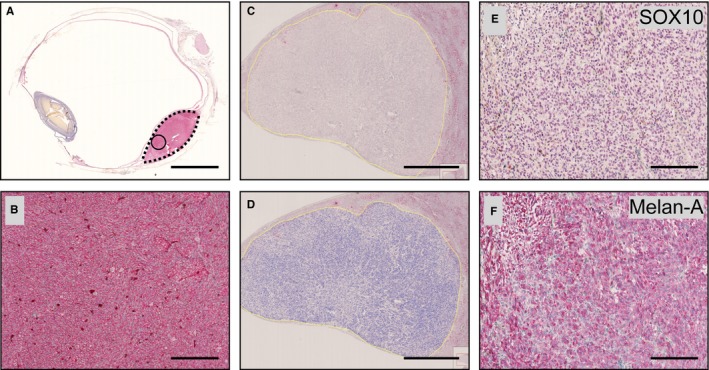
Examples of tissue regions and immunohistochemical markers. (A) In an enucleated eye, a primary tumor has been stained with TIGIT. A red chromogen is used. The circle illustrates a hot spot, chosen for its intensive staining in screening at 40×. In this area, the number of TIGIT positive cells per 3 hpf is counted at 400×. The dotted line illustrates how the tumor was outlined in analysis of full sections. (B) Magnification of the hot spot in a. Note that nearly all cells including tumor cells are positively stained. (C) A liver macrometastasis from the same patient, developed 4 y after the enucleation. A thin yellow line has been drawn to outline the metastasis. (D) In analysis with the digital image analysis software, most cells in the metastasis have been classified as negative for TIGIT (blue, in this case 72 TIGIT positive cells/mm^2^). (E and F) Magnifications of corresponding regions in the metastasis. Cell nuclei are stained with (E) SOX10 and cytoplasms with (F) Melan‐A. Scale bars, (A) 5 mm, (B) 300 μm, (C and D) 3 mm, (E and F) 250 μm. Hpf, High power fields

Six patients had developed metastases before death. The mean time from enucleation to detection of metastasis was 5.0 years (SD 3.0) and the mean survival after detection of metastasis was 0.4 years (SD 0.2). Four of 6 metastases were positive for SOX10 (2 with strong staining intensity, 2 with weak), and 5 of 6 were positive for Melan‐A (all with strong staining intensity). Morphologically, metastases were composed of spindle‐shaped cells growing in nests or storiform patterns, similar to the paired primary tumors (Figure 1). No metastasis was negative for both SOX10 and Melan‐A.

The remaining 4 patients had not developed metastases before death, and had other causes of death declared on their death certificates. These patients lived for 5.7, 11.2, 26.8 and 34.0 years after enucleation, with a mean of 19.2 years (SD 13.5).

Follow‐up was significantly longer for patients that did not develop metastasis before death. There were no other significant differences between the groups in age at enucleation, gender, tumor location, primary tumor thickness, diameter, primary tumor area on pathology slides or number of cells analyzed (Table 1).

**Table 1:** Characteristics of patients and tumors included in this study

	No metastasis	Metastasis	*P* a
*n* =	4	6	
Mean age at enucleation, years (SD)	56 (16)	69 (7)	0.14
Gender			1.0
Male, n	2	3	
Female, n	2	3	
Tumor location, n			1.0
Iris	0	0	
Ciliary body	0	0	
Choroid anterior to equator	0	0	
Choroid posterior to equator	4	6	
Extrascleral growth, n	0	0	1.0
AJCC T‐category, n			1.0
1	0	0	
2	4	6	
3	0	0	
4	0	0	
Mean tumor thickness, mm (SD)	5.5 (1.9)	5.2 (1.9)	0.34
Mean tumor diameter, mm (SD)	8.4 (2.6)	8.8 (2.8)	0.76
Mean primary tumor area, mm^2 ^(SD)^b^	24.6 (5.7)	20.8 (11.1)	0.55
Mean No. of cells, primary tumor (SD)^b^	272 264 (89 383)	206 816 (132 751)	0.18
Mean metastasis area, mm^2 ^(SD)^b^	—	134.7 (88.3)	
Mean No. of cells, metastasis (SD)^b^	—	1 424 568 (1 123 606)	
Previous treatment, n^c^	0	0	1.0
Follow‐up years, mean (SD)	19.2 y (13.5)	5.0 y (3.0)	0.03
Years metastasis to death, mean (SD)^d^	—	0.4 (0.2)	

Follow‐up was terminated at time of death.

a Student’s *t *test and Fisher’s exact tests were used for comparisons of continuous variables and categorical variables in two‐by‐two tables, respectively.

b As defined by digital image analysis.

c No patient had been treated with plaque brachytherapy, external beam radiation or transpupillary thermotherapy prior to enucleation.

d Equal variances not assumed. SD, Standard deviation.

## 4 IDO

Measured in hot spots, the mean number of IDO positive cells/mm^2^ was significantly higher in both metastatic and nonmetastatic primary tumors than in normal choroid tissue (*P* < 0.01), in metastases than in normal liver tissue (*P* < 0.01) and in primary tumors than in their paired metastases (*P* = 0.015), but not in metastatic versus nonmetastatic primary tumors (*P* = 0.079) or in nonmetastatic primary tumors versus nonpaired metastases (*P* = 0.43).

In full sections, the mean number of IDO positive cells/mm^2^ was significantly higher in both metastatic and nonmetastatic primary tumors than in normal choroid tissue (*P* < 0.01), in metastases than in normal liver tissue (*P* < 0.01), but not in metastatic versus nonmetastatic primary tumors (*P* = 0.45), in primary tumors versus their paired metastases (*P* = 0.13) or in nonmetastatic primary tumors versus nonpaired metastases (*P* = 0.11, Table 2).

**Table 2:** Number of IDO and TIGIT positive cells in different tissue types, defined in hot spots and across full tumor sections

	IDO+ cells/mm^2^, mean (SD)	*P* *	TIGIT+ cells/mm^2^, mean (SD)	*P* *	CD8+ cells/mm^2^, mean (SD)	*P* *
Hot spots						
Normal choroid	16 (21)		10 (6)		7 (7)	
Primary tumors, no metastasis	474 (346)	<0.01	1342 (617)	<0.01	301 (306)	0.065
Primary tumors, metastasis	1422 (885)	0.079	4695 (1334)	<0.01	838 (760)	0.22
Metastases	343 (158)	0.015	3831 (4381)	0.66	256 (361)	0.12
Normal liver	14 (21)	<0.01	4 (8)	<0.01	3 (3)	0.16
Full sections						
Normal choroid	9 (10)		3 (1)		2 (2)	
Primary tumors, no metastasis	322 (321)	<0.01	335 (252)	<0.01	95 (114)	0.11
Primary tumors, metastasis	720 (948)	0.45	2763 (844)	<0.01	217 (206)	0.32
Metastases	78 (94)	0.13	463 (371)	<0.01	19 (18)	0.04
Normal liver	2 (2)	<0.01	0 (0.5)	<0.01	1 (0.5)	0.058

* *P* for difference in mean from previous category. Paired samples *t* tests used for primary tumors vs. metastases.

**Figure 2:**
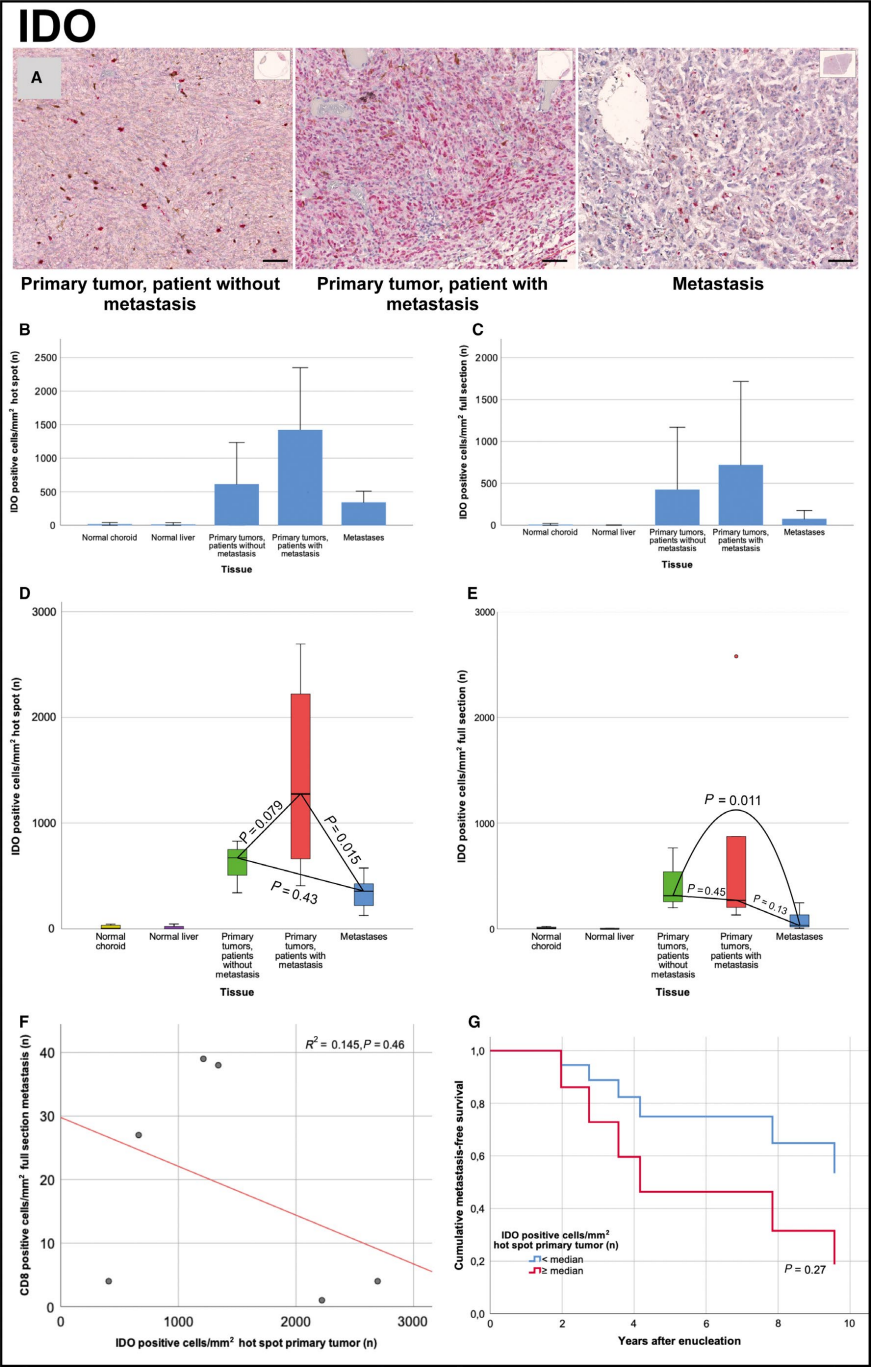
IDO expression in primary tumor and metastases. (A) Illustration of the density of IDO positive cells in primary tumors that did not metastasize (left), in primary tumors that metastasized (center) and in metastases (right). (B) Bar plot, mean number of IDO positive cells/mm^2^ in hot spots of normal choroid and liver tissue, in primary tumors that did not metastasize, in primary tumors that metastasized and in metastases. (C) Bar plot, mean number of IDO positive cells/mm^2^ in full sections of normal choroid and liver tissue, in primary tumors that did not metastasize, in primary tumors that metastasized and in metastases. (D) Box plot, IDO positive cells/mm^2^ in hot spots of primary tumors that metastasized versus primary tumors that did not metastasize (independent samples *t* test *P* = 0.079) and metastases (paired samples *t* test *P* = 0.013). (E) Box plot, IDO positive cells/mm^2^ in full sections of primary tumors that metastasized versus primary tumors that did not metastasize (independent samples *t* test *P* = 0.45) and metastases (paired samples *t* test *P* = 0.15). (F) Scatter plot with fit line, number of CD8 positive cells/mm^2^ in metastases versus number of IDO positive cells/mm^2^ in hot spots of primary tumors. Linear regression *R* ^2^ = 0.145, *P* = 0.46. (G) Cox regression cumulative metastasis‐free survival for patients with a hot spot density of IDO positive cells above and below the median. Hazard ratio above median = 2.7 (95% CI 0.5‐15.1, *P* = 0.27). Error bars represent 95% confidence interval. **°**=outlier. Scale bar 50 μm

In all types of tissue, the number of IDO positive cells/mm^2^ were higher than the number of CD8 positive cells/mm^2^, when measured in hot spots (*P* = 0.02) but not in full sections (*P* = 0.06). In linear regression, the number of CD8 positive cells/mm^2^ in metastases did not correlate significantly with the number of IDO positive cells/mm^2^ in primary tumor hot spots (*R* ^2^ = 0.145, *P* = 0.46). In Cox regression, the time dependent hazard for metastasis was not significantly increased for patients with a number of IDO positive cells/mm^2^ in primary tumor hot spots above the median (Hazard ratio IDO positive cells/mm^2^ above median = 2.7, 95% CI 0.5‐15.1, *P* = 0.27. Figure 2). In metastases, the number of IDO positive cells/ mm^2^ was not associated with survival (Hazard ratio IDO positive cells/mm^2^ above median in hot spots and full sections = 70.5, 95% CI 0.02 > 1000, *P* = 0.31).

## 5 TIGIT

Measured in hot spots, the mean number of TIGIT positive cells/mm^2^ was significantly higher in both metastatic and nonmetastatic primary tumors than in normal choroid tissue (*P* < 0.01), in metastases than in normal liver tissue (*P* < 0.01) and in metastatic than in nonmetastatic primary tumors (*P* < 0.01), but not in primary tumors versus their paired metastases (*P* = 0.66) or in nonmetastatic primary tumors versus nonpaired metastases (*P* = 0.30).

In full sections, the mean number of TIGIT positive cells/mm^2^ was significantly higher in all of: Primary tumors versus normal choroid tissue, metastases versus normal liver tissue, metastatic versus nonmetastatic primary tumors and in primary tumors versus their paired metastases (*P* < 0.01, Table 2). The exception was the difference between nonmetastatic primary and nonpaired metastases, which was not significant (*P* = 0.57).

**Figure 3:**
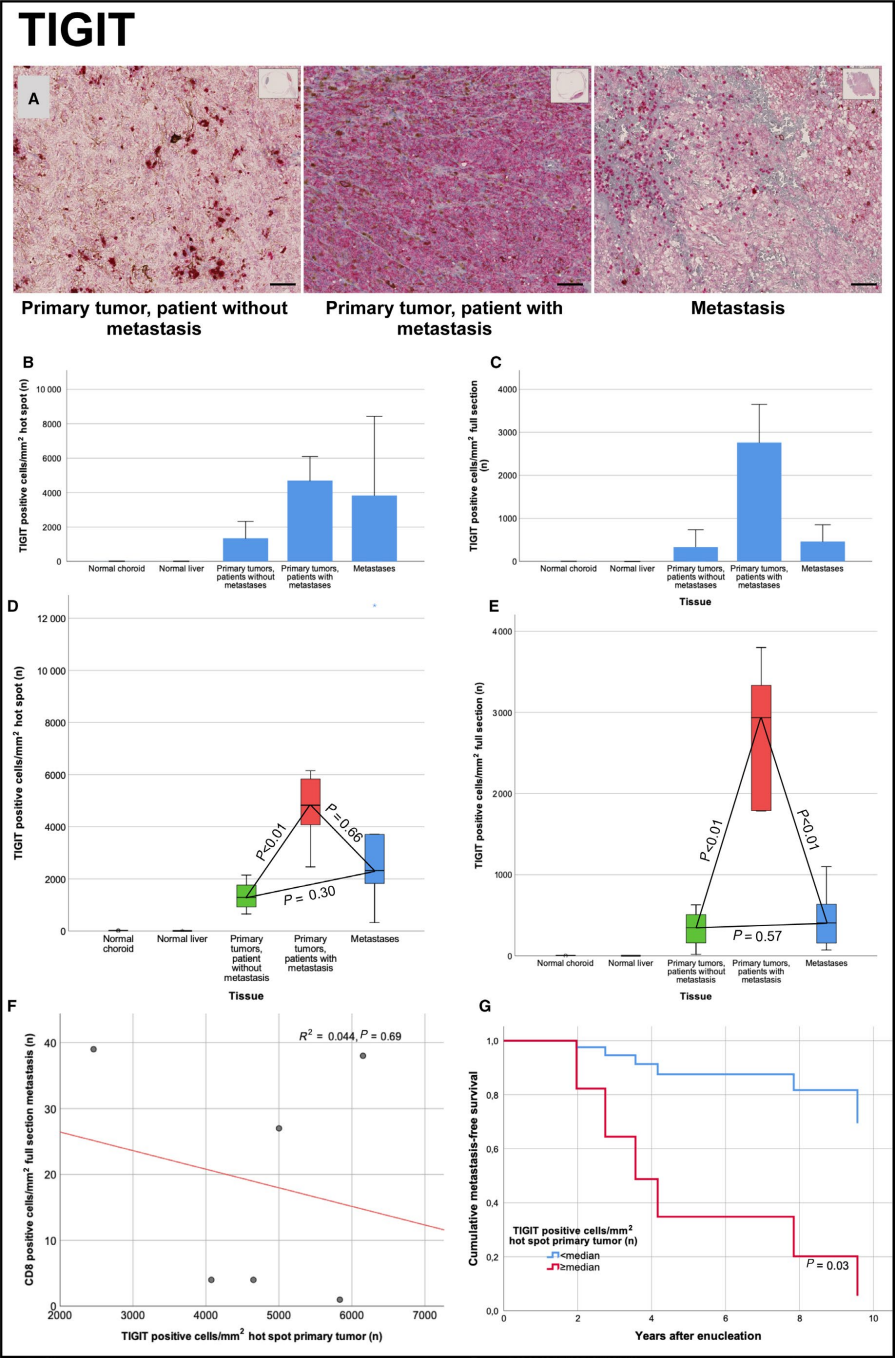
TIGIT expression in primary tumor and metastases. (A) Illustration of the density of TIGIT positive cells in primary tumors that did not metastasize (left), in primary tumors that metastasized (center) and in metastases (right). (B) Bar plot, mean number of TIGIT positive cells/mm^2^ in hot spots of normal choroid and liver tissue, in primary tumors that did not metastasize, in primary tumors that metastasized and in metastases. (C) Bar plot, mean number of TIGIT positive cells/mm^2^ in full sections of normal choroid and liver tissue, in primary tumors that did not metastasize, in primary tumors that metastasized and in metastases. (D) Box plot, TIGIT positive cells/mm^2^ in hot spots of primary tumors that metastasized versus primary tumors that did not metastasize (independent samples *t* test *P* = 0.079) and metastases (paired samples *t* test *P* = 0.043). (E) box plot, TIGIT positive cells/mm^2^ in full sections of primary tumors that metastasized versus primary tumors that did not metastasize (independent samples *t* test *P* = 0.45) and metastases (paired samples *t* test *P* = 0.15). (F) scatter plot with fit line, number of CD8 positive cells/mm^2^ in metastases versus number of TIGIT positive cells/mm^2^ in hot spots of primary tumors. Linear regression *R* ^2^ = 0.145, *P* = 0.46. (G) Cox regression cumulative metastasis‐free survival for patients with a hot spot density of TIGIT positive cells above and below the median. Hazard ratio above median = 2.7 (95% CI 0.5‐15.1, *P* = 0.27). Error bars represent 95% confidence interval.. *=extreme outlier. Scale bar 50 μm

The number of TIGIT positive cells/mm^2^ was higher than the number of CD8 positive cells/mm^2^ in all types of tissues, regardless if measured in hot spots or in full sections (*P* < 0.01). The number of CD8 positive cells/mm^2 ^was significantly higher in primary tumors than in their paired metastases measured in full sections (*P* = 0.04), but not in metastatic versus nonmetastatic primary tumors (*P* = 0.32, Table 2). In linear regression, the number of TIGIT positive cells/mm^2^ correlated with the number of IDO positive cells/mm^2^ in both hot spots (*R* ^2^ = 0.24, *P* < 0.01) and full sections (*R* ^2^ = 0.35, *P* < 0.01). The number of CD8 positive cells/mm^2^ in metastases did not correlate significantly with the number of TIGIT positive cells/mm^2^ in primary tumor hot spots (*R* ^2^ = 0.044, *P* = 0.69). In Cox regression, the time dependent hazard for metastasis was significantly increased for patients with a number of TIGIT positive cells/mm^2^ in primary tumor hot spots above the median (Hazard ratio TIGIT positive cells/mm^2^ above median = 7.9, 95% CI 1.0‐69.3, *P* = 0.03. Figure 3). In metastases, the number of TIGIT positive cells/ mm^2^ was not associated with worse (Hazard ratio TIGIT positive cells/mm^2^ above median in hot spots and full sections = 0.9, 95% CI 0.1‐9.2, *P* = 0.95).

## 6 DISCUSSION

In this study, the number of TIGIT positive cells was significantly higher in tumors from patients that eventually developed metastasis. Conversely, the half of our patients with highest density of TIGIT positive cells in their primary tumors at enucleation had an eightfold the rate of metastasis. The number of TIGIT positive cells was higher than the number of CD8 positive cells in all types of tissue examined, and correlated with the number of IDO positive cells. This signifies that it is not only the CD8 positive T cell infiltrate that previously has been identified in a third of primary UM that express these immune checkpoints.^7^ Indeed, based on our examinations of primary tumors and metastases, such expression was seen in tumor cells themselves. Expression of TIGIT was not significantly higher in metastases than in primary tumors from patients who did not develop metastasis, and in the case of IDO even lower, suggesting that the prognostic impact is greatest if checkpoints are expressed earlier in the course of the disease. In other words: Expression of immune checkpoints IDO and TIGIT in primary tumors is associated with subsequent development of metastasis. Once metastases have developed, the number of cells expressing IDO and TIGIT has no bearing on survival.

As shown by Singh *et al*,^17^ UM micrometastases can be seeded years before the primary tumor is diagnosed, and then remain dormant up to several decades before they start growing into clinically detectable lesions. This is the reason removal of the eye offer no survival advantage over eye‐preserving treatment for medium‐sized tumors, as shown in the Collaborative Ocular Melanoma Study.^18^ We hypothesize that early expression of immune checkpoints would promote the survival of such micrometastases, and therefore impede patient prognosis. This makes IDO and TIGIT highly interesting targets for adjuvant treatment, with a potential for better response rates than inhibition of PD‐1, PD‐L1, and CTLA4.

This study has several limitations. First, we have examined tissues from a very small number of patients leading to a low statistical power for detection of smaller but possibly important differences and to wide confidence intervals for our significant differences. Second, tissues and data were collected retrospectively at one center only. It is possible that other prognostic factors than immune checkpoints influence the rate of metastasis of the patients included. We sought to limit the impact of this by selecting patients and tumors with similar characteristics for our reference group. We have however not controlled for BAP‐1 expression, gene expression class or monosomy 3. Third, we used liver metastasis material obtained at autopsy, with a risk that the duration of cold ischemia has effected the immunoreactivity of the markers investigated.

In conclusion, immune checkpoints IDO and TIGIT have potential for being targets for adjuvant treatment in UM. Studies of larger cohorts that control for more factors, and preferably inclusion of UM patients in randomized trials of IDO and TIGIT inhibitors, are recommendable for drawing more definite conclusions.

## CONFLICT OF INTEREST

The authors made no disclosures.

## AUTHOR CONTRIBUTIONS

Gustav Stålhammar: Project conception, funding acquisition, design and execution of the experiments, data collection, statistical analysis, and writing of the manuscript. Stefan Seregard: Provision of clinical specimens, and writing–review and editing. Hans E. Grossniklaus: Project conception, funding acquisition, and writing–review and editing.
