# Supplementary material for: Expression of immune checkpoint receptors Indoleamine 2,3‐dioxygenase and T cell Ig and ITIM domain in metastatic versus nonmetastatic choroidal melanoma
Source: Cancer Med. 2019 Apr 16;8(6):2784–92. doi: 10.1002/cam4.2167 (PMC6558646; doi:10.1002/cam4.2167)
Supplement: Supplementary file 1 [file CAM4-8-2784-s001.docx]

| **Supplementary table 1. Digital image analysis settings used for all tumors.** | |
| --- | --- |
| **Setup parameters** | |
| Detection image | Haematoxylin OD |
| Requested pixel size | 0,5 μm |
| **Nucleus parameters** | |
| Background radius | 8 μm |
| Median filter radius | 0 μm |
| Sigma | 1,5 μm |
| Minimum area | 10 μm^2^ |
| Maximum area | 400 μm^2^ |
| **Intensity parameters** | |
| Threshold | 0.01 |
| Max background intensity | 2 |
| Split by shape | Yes |
| Exclude DAB | Yes |
| **Cell parameters** | |
| Cell expansion | 5 μm |
| Include cell nucleus | Yes |
| **General parameters** | |
| Smooth boundaries | Yes |
| Make measurements | Yes |
| **Intensity threshold parameters** | |
| Score compartment | Cytoplasm: DAB OD Mean |
| Threshold 1+ | 0.5 |
| Single threshold | Yes |
